# The Interstitial Fluid Compartment: Still Unrecognized or Biophysically Well Described?

**DOI:** 10.1002/cph4.70213

**Published:** 2026-07-07

**Authors:** Daniela Negrini, Giuseppe Miserocchi

**Affiliations:** ^1^ Department of Medicine and Technological Innovation (DIMIT) University of Insubria Varese Italy; ^2^ School of Medicine and Surgery University of Milano‐Bicocca Monza Italy

## Abstract

The proposal of a “new third bodily system for circulation of fluid” has been recently put forward. The present article sheds light on all available physiological knowledge concerning exactly this same space, defined as “interstitial compartment” as originally hypothesized by Ernest Starling about 130 years ago. We provide here a brief updated summary of the main morpho‐functional features and corresponding analysis concerning capillaries microfiltration, interstitial fluid dynamics, and lymphatic drainage.

## 1 Introduction

The present perspective article wishes to comment on the recently published paper (Benias et al. 2018) which was highly emphasized by the New York Times article on May 11th, 2026. Basically, the authors proposed the discovery of a “third bodily system for the circulation of fluids” in addition to the cardiovascular and lymphatic systems. We believe that this newly identified fluid space actually corresponds to what has been identified by Sir Ernest Starling back to 1896 (Starling 1896) as the “interstitial compartment” (IC). The latter corresponds to the extravascular, extracellular interstitial space placed in series between the systemic/pulmonary circulation and the lymphatic system that drains extravasated fluid to return it to the bloodstream. Fluid extravasation occurs down pressure gradients and is favored by a very low blood flow velocity in capillaries allowing bulk flows across the endothelium. IC might consist only of water and soluble molecules, as in the case of serosal fluids, or it might include a complex insoluble macromolecular structure (interstitial fibrillar matrix) through which free water percolates.

The interstitial fluid compartment has been deeply investigated from the standpoint of microfluidics focusing in particular on the mechanisms controlling the volume and composition of the extravascular fluid (Miserocchi and Beretta 2026; Negrini et al. 2026). The present article summaries the key points of the intravascular‐interstitial‐lymphatic system, focusing on interorgan functional differences.

## 2 Structure and Function

**FIGURE 1:**
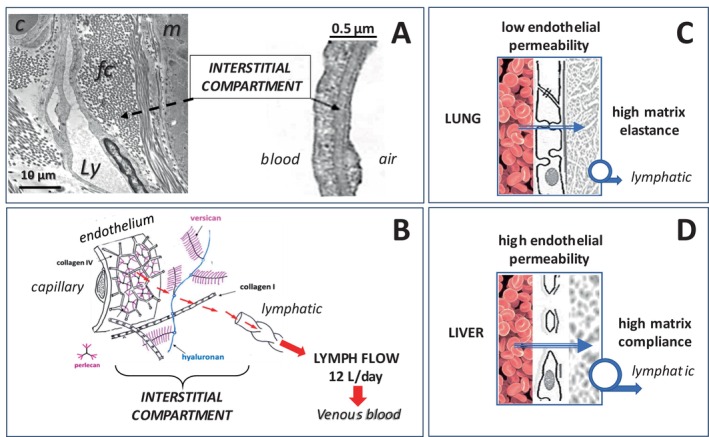
(A) TEM images of the interstitial compartments in the diaphragm (left) and in the lung. (B) Schematic drawing of transcapillary water fluxes through the endothelial basement membrane and interstitial compartment to reach the lymphatic vessel. The main structural molecules are indicated. (C) Three compartments model depicting fluid extravasation from lung capillaries into surrounding matrix down to lymphatic outlet. The low endothelial permeability and high matrix elastance minimize trans‐endothelial flux and lymphatic flow. (D) Opposite features, as in the liver, lead to high filtration rate and lymphatic drainage.

Panel A in Figure 1 allows us to characterize IC on morphological ground and to compare tissues with remarkably different water fluxes and contents. In the left panel, in a skeletal muscle section (diaphragm) *c* refers to a capillary, *fc* is the dense mesh of fibrillar collagen I, *m* is a skeletal muscle cell, and *Ly* is an initial lymphatic channel. On the right panel, a transverse section of the lung air‐blood barrier highlights its remarkable subtlety as opposed to the muscle. Panel B depicts schematically the morpho‐functional model of capillary‐to‐tissues fluid exchanges. Based on available evidence (Miserocchi et al. 1990, 1991; Michel 1997), at variance with the original hypothesis by Starling (1896), no fluid reabsorption basically occurs at the venular end of the capillary in the large majority of tissues. In fact, trans‐endothelial filtered fluid drains through the complex interstitial matrix, as depicted in Figure 1B, to be eventually drained into lymphatics. Noteworthy, in physiological conditions the overall amount of lymph flow to reach the venous blood is of the order of about 12 L/day, about four times plasma volume. Lack of lymphatics occurs in avascular tissues, such as epidermis, cartilage, cornea and the inner layer of large arteries, as well as in vascularized brain parenchyma, retina, bone marrow (Schmid‐Schönbein 1990); in these organs microvascular filtration is minimal and reabsorption occurs at the venular side of the capillaries.

Panels C and D allow us to compare how trans‐endothelial water fluxes and interstitial fluid volume may considerably vary among tissues, depending on the water permeability of the endothelial barrier and of the interstitial matrix that provides further viscous resistance to water flux. In the lung (Figure 1C), water permeability is extremely low (Taylor et al. 1982) due to continuous endothelium (Conforti et al. 2002) and a highly rigid fibrillar interstitial matrix (Miserocchi et al. 1990, 1991). This setting guarantees the extreme thinness of the air‐blood barrier to ensure optimal gas exchanges. The opposite occurs, as an example, in the liver (Figure 1D) due to fenestrated endothelium, discontinuous basement membrane, and a highly compliant matrix structure (Aukland and Reed 1993).

**FIGURE 2:**
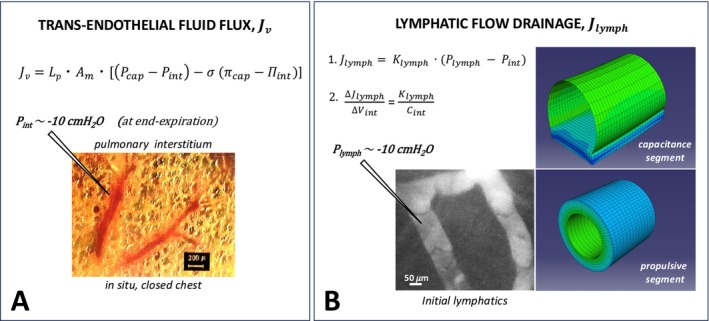
(A) Trans‐endothelial fluid fluxes (*J*_*v*_) are described by the revised Starling equation. Parameters are defined in the text. Lower panel: Microphotograph of the transpleural micropuncture recording of peri‐microvascular interstitial pressure (Miserocchi et al. 1990). (B) Constitutive equations for lymphatic flow. Equation 1 defines the gain of lymph flow expressed as a product of pressure gradients times lymphatic conductance. Equation 2 expresses the efficiency of the drainage, where ∆ *V*_int_ and *C*_int_ represent, respectively, a unit of drained interstitial fluid volume and the compliance of the interstitial matrix.

Figure 2 recalls the physical laws governing microfluidics. Panel A refers to transmembrane water fluxes, based on nonequilibrium thermodynamics (Kedem and Katchlasky 1958; Michel 1997). The trans‐endothelial fluid bulk flow (*J*_v_) depends upon the balance between the hydraulic (*P*) and colloidosmotic (π) pressures in the capillary lumen (cap) and in the perivascular interstitial space (int), according to the equation:

$$J_{V}=L_{p}\cdot A_{m}\cdot \left[\left(P_{\mathrm{cap}}-P_{\mathrm{int}}\right)-\sigma \cdot \left(\pi_{\mathrm{cap}}-\pi_{\mathrm{int}}\right)\right]$$

where *L*_*p*_ is the endothelial hydraulic conductivity and *A*_*m*_ its surface area available for fluid exchange. The reflection coefficient of the endothelium to plasma proteins, *σ*, is a function of the solute to endothelial pore radii ratio: *σ*→1 for large solutes being reflected by the membrane wall, while *σ*→0 for smaller, easily permeable solutes. Accordingly, based on the *σ* value, the protein concentration of the interstitial fluid and corresponding π_int_ may greatly vary. For *σ*→1, as in the lung, π_int_ is close to zero; conversely, for *σ*→0, as in hepatic sinusoids, π_int_ is similar to plasma values (Curry 1984). Such a biophysical model is further complicated when analyzing multiple compartments placed in series, as in the case of the air‐blood barrier that includes two membranes (endothelium and epithelium) and three fluid compartments (plasma‐interstitial compartment—alveolar lining).

Interestingly, one may recall that the functional importance of the tissue architecture and forces in promoting fluid and solute fluxes and distribution within the extracellular compartment has been elegantly analyzed accounting for single vessel permeabilities, dimensions, and pressures (Guidoboni et al. 2021).

## 3 Interstitial Pressure Measurements

Measurements of all terms and coefficients in the above equation have always been a challenge. Guyton was the first to focus on *P*_int_ and π_int_, key variables being of difficult experimental access (Guyton 1963). Using a complex though invasive approach Guyton studied different tissues, including the lung (Guyton et al. 1971), concluding for the existence of a subatmospheric pressure in the IC of most tissues. A major step forward was made by applying, in several organs, the micropuncture technique to directly record *P*_int_, as well as the “wick” technique to measure π_int_ (Aukland and Reed 1993). These approaches allowed to directly measure (Figure 2A, lower panel) *P*_int_ in the lung physiologically expanded in the closed chest (Miserocchi et al. 1990, 1991; Miserocchi, Negrini, et al. 1993) and π_int_ in the peri‐microvascular pulmonary interstitial compartment (Negrini et al. 2001).

Interestingly, both the pulmonary interstitial compartment and the pleural space are characterized by highly sub‐atmospheric pressure and minimum interstitial volume (Miserocchi, Venturoli, et al. 1993). This analogy led us to put the question concerning how a physiological steady state condition could be maintained despite a continuous and variable capillary microvascular filtration to assure the functionally optimal tissue hydration. Concerning this last aspect, the solution of the transcapillary flow equation in the lung reveals a net continuous minimal microvascular filtration towards the interstitial compartment as low as 1 × 10^−4^ mL·cm−^2^·day^−1^, a value about 10^−5^ fold with respect to respiratory gas diffusive fluxes (Miserocchi 2009).

The disrupture of the equilibrium between trans‐endothelial fluid filtration and lymphatic drainage coupled to the deterioration of the matrix mechanical properties invariably leads to tissue oedema. In this regard, considering the development of lung edema, an important contribution had been provided by the studies carried on by Bhattacharya's group, that extensively measured pulmonary interstitial pressure in isolated perfused dog lung exposed to various degrees of lung inflation and perfusion (Bhattacharya et al. 1984, 1989; Glucksberg and Bhattacharya 1991; Mehta et al. 2004).

## 4 Lymphatic Drainage

Panel 2B presents some aspects of the lymphatic fluid drainage. The lymphatic vasculature is a closed unidirectional network that embryologically belongs to the cardiovascular system. By continuously removing fluid from the interstitial compartment and returning it to the venous bloodstream, the lymphatic system maintains the physiological hydration in the tissues. The interstitial fluid is driven first into initial lymphatics, tiny vessels delimited by a single layer of endothelial cells with a discontinuous basal membrane (Schmid‐Schönbein 1990). As the initial lymphatic endothelium offers no effective sieving (*σ* = 0) to large macromolecules (and even cells) (Guyton et al. 1975), the colloidosmotic contribution is nil and lymphatic fluid flux (*J*_lymph_) is thus given by Equation 1 in Panel 2B:

(1) $$J_{\text{lymph}}=K_{\text{lymph}}\cdot \left(P_{\text{lymph}}-P_{\mathrm{int}}\right)$$

where *P*_lymph_ is the hydraulic pressure in the initial lymphatics (Miserocchi et al. 1989; Negrini and Del Fabbro 1999; Moriondo et al. 2005) and *K*_lymph_ is lymphatic conductance. The lower left panel in Figure 2B refers to an actual micropuncture measurement of *P*_lymph_ between two intraluminar valves (Negrini and Del Fabbro 1999).

Pressure gradients sustaining lymph formation rely on a combination of intrinsic lymphatic muscle contraction, external skeletal or cardiac muscle contraction with the necessary contribution of the unidirectional valves. The tensile stresses developing in the lymphatic walls have been modeled by a finite element analysis leading to identification of capacitance and propulsive segments placed in series (Figure 2, upper and lower right panels). Interestingly *K*_lymph_ is not constant and may increase several fold (Gee and Spath 1980; Taylor 1981) exploiting the action of the so called anchoring filaments, that exert an outward pulling action on the lymphatic wall (Leak 1970; Schmid‐Schönbein 1990).

The lymphatic draining role has also been carefully analyzed by quantifying the contributions of the collecting lymphatics in the mesenteric interstitium to the movement of fluid and solute both into and out of the vascular and interstitial compartment to finally reach the lymphatic lumen (Huxley and Scallan 2011). The reported measures of hydrostatic pressure data are compatible with a viable lymphatic function creating a relatively subatmospheric interstitial pressure (Huxley and Scallan 2011).

The “efficiency” of lymphatic removal can be defined (Figure 2B, Equation 2) by the ratio $\frac{∆J_{\text{lymph}}}{∆V_{\mathrm{int}}}=\frac{K_{\text{lymph}}}{C_{\mathrm{int}}}$ (Miserocchi 2009), where *C*_int_ is interstitial matrix compliance and $∆V_{\mathrm{int}}$·is the unit of drained interstitial fluid volume. Hence, efficiency is high as long as *C*_int_ remains low as in the normal intact lung. When the increase in microvascular permeability and the associated matrix degradation (Negrini et al. 1996) cause an increase in *C*_int_ the efficiency of lymphatic system is compromised, inevitably leadings to tissue oedema. Such condition, that may be tolerable in some organs like for example the subcutaneous tissue, may be deadly harmful in the lung (Mazzuca et al. 2016).

## 5 Conclusions

In summary, the capillary filtration—lymphatic drainage is a complex and delicate capacitance‐to‐resistance system placed in series. The filtration‐absorption balance is variable among organs depending on their function. Mechanical compartment properties as well as filtration/drainage resistive features are critical in maintaining fluid homeostasis. Lymphatics act as a passive negative feedback control able to offset any increase in interstitial fluid volume (Miserocchi 2009).

## Funding

The authors have nothing to report.

## Disclosure

Permission to Reproduce Material from other Sources: The Figures have been either modified with respect to the already published originals or have never been published elsewhere.

## Ethics Statement

The authors have nothing to report.

## Consent

The authors have nothing to report.

## Conflicts of Interest

The authors declare no conflicts of interest.

## Data Availability

The data that support the findings of this study are available from the corresponding author upon reasonable request.
